# Health care input constraints and cost effectiveness analysis decision rules

**DOI:** 10.1016/j.socscimed.2018.01.026

**Published:** 2018-03

**Authors:** Pieter van Baal, Alec Morton, Johan L. Severens

**Affiliations:** a Erasmus University Rotterdam, Erasmus School of Health Policy & Management, Rotterdam, The Netherlands; b University of Strathclyde, Department of Management Science, Glasgow, United Kingdom

**Keywords:** Cost-effectiveness analysis, Human resource constraints, Decision rules, Health care input constraints, Opportunity costs, Eye-care services, LMIC

## Abstract

Results of cost effectiveness analyses (CEA) studies are most useful for decision makers if they face only one constraint: the health care budget. However, in practice, decision makers wishing to use the results of CEA studies may face multiple resource constraints relating to, for instance, constraints in health care inputs such as a shortage of skilled labour. The presence of multiple resource constraints influences the decision rules of CEA and limits the usefulness of traditional CEA studies for decision makers. The goal of this paper is to illustrate how results of CEA can be interpreted and used in case a decision maker faces a health care input constraint.

We set up a theoretical model describing the optimal allocation of the health care budget in the presence of a health care input constraint. Insights derived from that model were used to analyse a stylized example based on a decision about a surgical robot as well as a published cost effectiveness study on eye care services in Zambia.

Our theoretical model shows that applying default decision rules in the presence of a health care input constraint leads to suboptimal decisions but that there are ways of preserving the traditional decision rules of CEA by reweighing different cost categories. The examples illustrate how such adjustments can be made, and makes clear that optimal decisions depend crucially on such adjustments.

We conclude that it is possible to use the results of cost effectiveness studies in the presence of health care input constraints if results are properly adjusted.

## Highlights

• Health care input constraints affect cost effectiveness analysis decision rules.
• However, if ICERS are adjusted traditional CEA decision rules can be preserved.
• In specific cases it is possible to adjust conventional ICERs.
• ICER adjustments require detailed reporting of cost effectiveness results.

## 1 Introduction

Health economic evaluations aim to inform decision-making about new health care technologies in order to make more efficient use of scarce resources (Drummond et al., 2015). Although the starting point for economic evaluations is that resources are scarce and thus that there is a limit to what can be spent on health care, other constraints besides the health care budget might be relevant in this context (Hauck et al., 2016, Vassall et al., 2016). Consequently, while in the long run many constraints can (in theory) be resolved by a more efficient allocation of resources, ignoring such constraints in economic evaluation might seriously hamper the usefulness and credibility of economic evaluations in health care decision making (Eddama and Coast, 2008). In the short-run, there are numerous constraints involved, consisting of supply-side (e.g. workforce shortages), demand-side (e.g. obstacles of access to healthcare) and healthcare system constraints (e.g. regulatory constraints). One particular type of constraints relevant for economic evaluations are constraints related to health care inputs. Constraints related to health care inputs usually are an indicator of market failure which may be caused by the fact that markets for health care inputs are heavily regulated with the aim to solve problems of information asymmetry (Dranove, 2011, Nicholson and Propper, 2011, Scott Morton and Kyle, 2011). As a result, markets for health care inputs are often characterized by monopsony buyers and/or monopoly producers. Monopoly producers usually force prices to be too high (which is often the case in medicines) and monopsony employers may force prices of labour to be too low. Consequently, the market price or market salary of inputs for economic evaluations do not reflect true opportunity costs which violates the standard model of cost effectiveness analysis (Drummond et al., 2015).

While previous research has focused on the impact of constraints on estimates of costs and benefits of health care interventions (Hauck et al., 2016, Vassall et al., 2016) it is not always realized that such constraints may also influence how optimal decisions conditional on those estimates should be made. The default decision rules of cost effectiveness analyses where cost effectiveness ratios are compared to a threshold level of cost effectiveness, are derived from an optimization problem with only one constraint: the health care budget (Karlsson and Johannesson, 1996, Weinstein and Zeckhauser, 1973). The theory behind this is that most constraints can be resolved and the only relevant constraint in the long run is the health care budget. However, as some constraints can be persistent and difficult to resolve in some settings, the rather abstract long run view typically taken in cost effectiveness in which the only constraint is the health care budget might not be the most appropriate view (Adang, 2008, Van de Wetering et al., 2012). Often, decision makers have to take many constraints as given and do not have the discretion to relax those constraints (Adang, 2008, Eddama and Coast, 2008, Hauck et al., 2016, Van de Wetering et al., 2012). For instance, decision makers making decisions about technologies within a single disease programme often need to make use of highly specific health care inputs which might be constrained and decisions about expanding or contracting certain health care services might crucially depend on the availability of such constrained health care inputs. For example, treatment for anxiety and depression consists mainly of pharmacological treatments and talking therapies. In many settings, human resources (ie therapists) are constrained, since training therapists takes time and money (and often therapists may have to pay the costs of their own training) (Haby et al., 2004). Even if talking therapies might seem more cost-effective in some circumstances, in the presence of a constraint on the number of therapists it might be more efficient to provide pharmacological treatments. More generally, in low and middle income countries (LMIC) there is often a lack of supply of skilled doctors and nurses which might influence costs and health effects of delivering a particular health care technology (Fulton et al., 2011, World Health Organization, 2006). Increasing the size and skills of the workforce is often not that easy (Wyss, 2004) and raising wages to increase the workforce in low income countries might have limited success as it is difficult to compete with wages in Western countries (Robinson and Clark, 2008). In these settings, human resource constraints limit the usefulness of CEA studies for decision makers as applying the standard decision rules could result in suboptimal decisions.

In case of multiple constraints, the default decision rules of cost effectiveness do not apply anymore and decision rules become more complex (Stinnett and Paltiel, 1996). As a solution to this, some studies have advocated the use of mathematical programming to arrive at an optimal allocation of resources in the presence of multiple constraints (Epstein et al., 2007, Feenstra et al., 2011, Stinnett and Paltiel, 1996). In these studies, numerous constraints were considered varying from demand and supply constraints to equity constraints. A drawback of mathematical programming is that the analytical capabilities for these techniques are substantial and that it is difficult to translate insights from such studies, in which usually lots of interventions are included, to simple cost effectiveness studies where only a few interventions are compared and central outcomes expressed in incremental cost effectiveness ratios (ICER). The goal of this paper is to show how health care input constraints may affect the decision rules of cost effectiveness analysis and to illustrate how results of CEA studies can be interpreted and used in case a decision maker faces a health care input constraint. As a starting point we take the most popular form of economic evaluation in which ICERS of interventions are estimated from a health care perspective and compared to a threshold level of cost effectiveness. The results of such incremental analyses are used to inform decision makers who usually have to take many constraints as given. Note that our analyses is closely related to the literature in cost benefit analyses that deals with estimating shadow prices in the presence of market failures (Drèze and Stern, 1990). Also note that in this paper a health care perspective is taken where the health care budget is assumed exogenous to the decision problem (Meltzer, 1997, van Baal et al., 2016). However, insights that we gain in this paper also apply if the perspective is broadened to a wider societal perspective.

## 2 Stylized example

To motivate the analysis, consider the following stylized but realistic example. A regional health authority at some time in the near future is planning investment in a fleet of surgical robots for some high-volume operation (say knee replacements). The robots require capital investment but will reduce inpatient admissions and outpatient attendances, thus saving on staff time (Barbash and Glied, 2010, Hughes et al., 2016). The health authority is constrained in terms of medical expertise. (Perhaps this may because the country's medical schools do not train enough doctors and the country has historically made up the shortfall by importing doctors from low-income countries, but popular resistance to immigration now makes this impossible: but these details need not concern us.) The health authority has two options for how they conduct operations:

• Option T (traditional, non-robot supported surgery) produces operations at unit cost of $10k of which $8k consists of spending on human resources;
• Option R (robot supported surgery) produces operations at a unit cost of $20k of which $5k consists of spending on human resources.

**Fig. 1:**
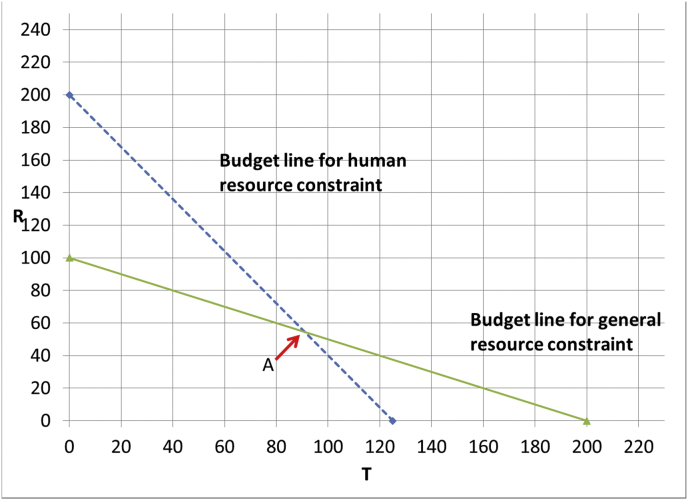
Budget lines for interventions T and R given a total health care budget of 2m dollars and a human resource budget of 1m dollars.

Assume that operations produced by the traditional and robot-supported surgery are comparable in terms of quality of life, and specifically, that both produce 1 QALY. Assume also that the workforce can perform all said interventions, and that there is a waiting list: hence there is no shortage of patients to treat. If we have a health care budget of $2m and we are not concerned about the shortage of medical staff we would simply invest the whole budget in T which results in 200 operations and hence 200 QALYs and $1.6m would be spent on human resources. Now suppose the shortage of doctors means that only $1m can be spent on human resources. What would then be the optimal allocation of resources? Spending the entire budget on T is not an option anymore because it is only possible to provide 1,000,000/8000 = 125 patients T leaving a slack in the budget of $750k. However, spending the entire budget on R is possible but results in even fewer operations (100). So, here the optimal solution is a mix of T and R. As is well-known (Crown et al., 2017), this can be found graphically (see Fig. 1). In this figure, the axes represent the number of units of T and R purchased. As both T and R produce the same number of operations and hence of QALYs, the total number of QALYs produced at point x is simply the Manhattan distance between x and the origin (i.e. the number obtained by counting along the T axis from the origin and then up in the vertical direction until x is reached). The feasible region is the area to the left of both constraints with the solid line representing the general constraint and the dashed line representing the human resource constraints. The optimal solution is the point A which corresponds to a mix of approximately 55 patients treated with the robot and about 90 patients being treated traditionally leading to 3200/22 or about 145 QALYs.

Now suppose that a new technology arrives on the marketplace – a new generation robot which partially automates surgery (as opposed to merely supporting it) and so reduces the need for surgeon time. This robot, R2, is more costly than R – it costs $25k per operation, but only $1k of this is spent on human resources. How should we assess how much this technology is worth for investment purposes?

**Fig. 2:**
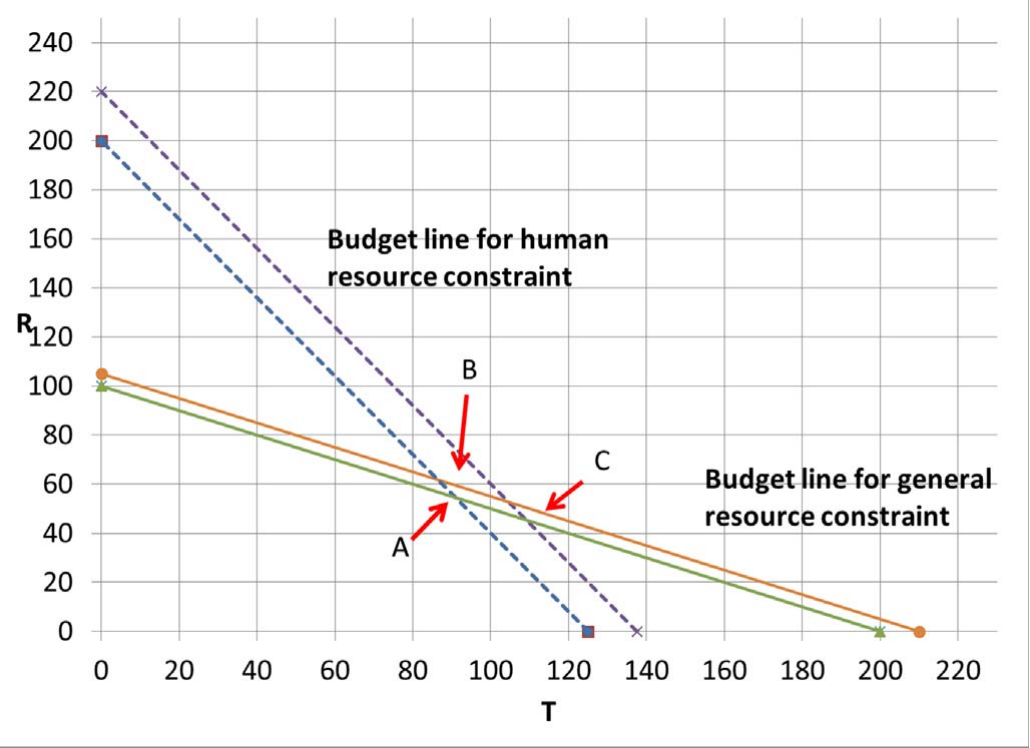
Perturbing the general and human resource specific budget constraints one at a time changes the optimum solution.

We can also address this question graphically. To do this, we can relax each of the budget constraints in turn by $100k and see what happens to number of QALYs produced. As shown in Fig. 2, the number of QALYs produced increases by 60/22 (to 3260/22 QALYs at point B) and 200/22 (to 3400/22 QALYs at point C) for the general and human resource specific budget constraints specifically. These numbers are a measure of the health displaced by the consumption of the general budget and also the human resource specific budget at the current optimum.

What does this tell us about the cost-effectiveness of investing in R2? At the margin, each operation conducted by R2 will reduce the general budget by $25K and hence cost 15/22 QALYs and the human resource budget by $1K and hence cost 2/22 QALYs. Hence, each operation conducted by the automating robot produces 1 QALY – but results only in the displacement of 17/22 ((15+2)/22) QALYs, and so we can conclude that investing in the R2 robot is worthwhile.

Our point here is that this is an environment where human resource constraints compel the implementation of a mix of interventions. When making decisions about new technologies in such an environment, decision makers should take account of the impact on both the general (money) budget and also on the availability of this skilled staff. In the remainder of the paper we present some formal analysis of this problem, and show how the optimal decision rule in this context can be seen as a generalisation of the familiar decision rule of checking whether the ICER is above or below a threshold. We show how our analysis can be applied in the stylized example of this section, and produces the same results, though by a more direct route.

## 3 Theoretical framework

To formally derive decision rules for cost effectiveness analysis in the presence of a health care input constraint we will formally derive them from an optimization problem. Here, we will make a distinction between spending on two different health care inputs, which allows us to express constraints in terms of health spending. Note that health care input constraints can also be modelled by capacity constraints using volume indicators (e.g. number of doctors/nurses or time available from doctors/nurses) (Feenstra et al., 2011). However, as will become clear, by expressing the health care input constraint in terms of spending it is easier to make the link with standard cost effectiveness studies and the way in they are currently presented and used.

Consider two different patient populations and let *i* and *j* indicate the interventions for these two patient groups (both *i* and *j* are assumed to be greater or equal to zero). Health (denoted *h*) and health care spending (denoted *c*) are a function of these interventions. The health care budget (denoted *B*) is the sum of spending on two types of health care inputs (denoted *x* and *y*) and spending on interventions *i* and *j* can be broken down by spending on these inputs ($c\left(i,j\right)=c_{x}\left(i,j\right)+c_{y}\left(i,j\right)$). As a starting point, let us assume that the health care budget can be spent freely on both health care inputs. To solve this optimization problem we will define a Lagrangian function L:

(1) $$Max\;L=h\left(i,j\right)+\lambda \left[B-c_{x}\left(i,j\right)-c_{y}\left(i,j\right)\right]$$

First order conditions for interventions *i* and *j* of this optimization problem are:

(2) $$\frac{\partial L}{\partial i}=0\;\to \;\frac{\partial h}{\partial i}=\lambda \left(\frac{\partial c_{x}}{\partial i}+\frac{\partial c_{y}}{\partial i}\right)$$

(2a) $$\frac{\partial L}{\partial j}=0\;\to \;\frac{\partial h}{\partial j}=\lambda \left(\frac{\partial c_{x}}{\partial j}+\frac{\partial c_{y}}{\partial j}\right)$$

These first order conditions can be rearranged to obtain expressions which resemble the standard decision rules of cost effectiveness:

(3) ∂cx∂i+∂cy∂i∂h∂i=∂cx∂j+∂cy∂j∂h∂j=1λ

Here $\frac{\frac{\partial c_{x}}{\partial i}+\frac{\partial c_{y}}{\partial i}}{\frac{\partial h}{\partial i}}$ and $\frac{\frac{\partial c_{x}}{\partial j}+\frac{\partial c_{y}}{\partial j}}{\frac{\partial h}{\partial j}}$ can be interpreted as the ICERs for spending on interventions *i* and *j* and 1/λ the threshold level of cost-effectiveness (often referred as *k* (Claxton et al., 2010, Claxton et al., 2011)) which makes sense as λ equals the shadow price of the health care budget: the amount of QALYs that can be obtained by increasing the budget with one unit. Equation (3) makes clear that at the optimum the marginal cost effectiveness should be equal in both patient groups. The model summarized in equation (1) also provides us insights in the allocation of the two health care inputs. In case the health care budget can be spent freely a change in spending on input *x* is compensated by a change in spending on input *y*: $dc_{x}=-dc_{y}$. As a consequence, at the optimum the marginal returns to spending on input *x* should equal the marginal returns to spending on input *y*:

(4) $$\frac{dh}{dc_{x}}=\frac{dh}{dc_{y}}$$

This is equivalent to saying that the marginal cost-effectiveness of all health care inputs should be equal if the health care budget can be spent freely on these different health care inputs. To better understand the nature of health care input constraints and the impact on the decision rules of cost effectiveness let us drop the assumption that the total health care budget can be spent freely. Suppose now that spending health care input *x* is constrained and spending on *y* is not constrained as long as total health spending does not exceed the budget. For instance, we can think of spending on *x* as spending on constrained human resources (denoted $B_{x}$) such as for instance spending on doctors which is constrained due to a shortage of doctors. The Lagrangian function *L* can now be written as:

(5) $$Max\;L=h\left(i,j\right)+\lambda \left[B-c_{x}\left(i,j\right)-c_{y}\left(i,j\right)\right]+\lambda_{x}\left[B_{x}-c_{x}\left(i,j\right)\right]$$

Subject to suitable continuity and convexity assumptions, first order conditions for an optimum are now:

(6) $$\frac{\partial L}{\partial i}=0\;\to \;\frac{\partial h}{\partial i}=\lambda \left(\frac{\partial c_{x}}{\partial i}+\frac{\partial c_{y}}{\partial i}\right)+\lambda_{x}\frac{\partial c_{x}}{\partial i}$$

(6a) $$\frac{\partial L}{\partial j}=0\;\to \;\frac{\partial h}{\partial j}=\lambda \left(\frac{\partial c_{x}}{\partial j}+\frac{\partial c_{y}}{\partial j}\right)+\lambda_{x}\frac{\partial c_{x}}{\partial j}$$

The big difference with equation (2) is that there is an additional lamda ($\lambda_{x}$) which can be interpreted as the shadow price of the constrained health care inputs within the health care budget. The shadow price of the health care budget can now be interpreted as the shadow price of spending on *y.* Furthermore, if the total health care budget cannot be allocated without restrictions it does not necessarily follow that these shadow prices are equal. This gives the situation in which health care input constraints apply:

(7) $$\lambda_{x}>\lambda \;\text{implying}\;\frac{dh}{dc_{x}}>\frac{dh}{dc_{y}}\;\text{and}\;\frac{dc_{x}}{dh}<\frac{dc_{y}}{dh}$$

Health care input constraints can thus be understood as meaning that the marginal benefits of spending on those inputs are higher than those of spending on other health care inputs. In other words, we can only speak of health care input constraints if reallocating spending from other health care inputs to the constrained inputs would result in health gains. Note that our definition of HRC is distinct from that of allocative inefficiency. Conditional on the human resources available it may well be the case that the production of health is efficient. If $\lambda_{x}>\lambda$ it would be desirable to reallocate the total health care budget towards more spending on *x* and less on *y* which reduces $\lambda_{x}$ and increases λ. However, in practice this might not be possible because of a shortage on specific health care inputs. As a consequence, cost-effective interventions that require a lot of those inputs cannot be implemented forcing the health care budget to be spent on interventions that seem less cost-effective but require less of the constrained inputs. This has clear implications for the decision rules of cost effectiveness analyses. In the presence of an input constraint ICER expressions become more complicated and look like the following:

(8) (1+λxλ)∂cx∂i+∂cy∂i∂h∂i=(1+λxλ)∂cx∂j+∂cy∂j∂h∂j=1λ

Equation (8) illustrates that costs in the ICER need to be expressed in one common numeraire by weighing the costs of the constrained input more heavily than costs of the unconstrained health care inputs. Note that (8) collapses to equation (3) in case there is no input constraint as $\lambda_{x}$ equals zero in that case. The implication of (8) is simple: standard CE ratios give a biased estimate of cost effectiveness with the bias becoming stronger a) the more constrained inputs an intervention uses b) the bigger the difference in shadow prices between the different health care inputs. Practically speaking, erroneously applying (3) instead of (8) may lead to different ordering of interventions in terms of cost effectiveness, potentially wrong decisions and counterintuitive results (see next section). For instance, an increase of the budget on other inputs than the constrained input leads to a substitution from interventions that seem more cost effective according to (3) but which require more constrained inputs towards interventions that require fewer constrained inputs but that have a higher ICER as estimated by (3).

A difficulty is that equation (8) suggest that two thresholds are needed, while in practice it is already challenging to estimate one threshold: the threshold of the overall health care budget. However, there is a way around this by looking at the linear special case of the model in equation (5) which assumes constant returns to scale and perfect divisibility. At an optimum in which a mix of interventions are used and at which demand constraints on both interventions are non-binding, (8) specialises as follows:

(9) $$Max\;L=i+j+\lambda \left[B-s\;i-t\;j\;\right]+\lambda_{x}\left[B_{x}-p\;i-q\;j\right]$$

Here, in equation (9) interventions *i* and *j* produce one unit of health at constant marginal costs: *s* and *t* denote the total per health unit costs of intervention *i* and *j* while *p* and *q* denote the constrained health care input cost per health unit. In this case (6) and (6a) collapse to:

(10) $$\frac{\partial L}{\partial i}=0\;\to \;1=s\;\lambda +p\;\lambda_{x}$$

(10a) $$\frac{\partial L}{\partial j}=0\;\to \;1=t\;\lambda +q\;\lambda_{x}$$

which allows to express one threshold as a function of the other and calculate the ratio of thresholds as needed in (8):

(11) $$\frac{\lambda_{x}}{\lambda }\;=\frac{s-t}{q-p}\;$$

Equation (11) can be used to re-analyse and interpret CEA studies in the presence of a health care input constraint given estimates of *p, q, s* and *t*. Applying our model to the example of the previous section, *s* = 20, *t* = 10, *p* = 5 and *q* = 8, hence the expression given in equation (11) equals 10/3. It can be seen that this is indeed equal to the ratio of the marginal valuation of the human resource specific (200/22) and general (60/22) budget constraints as calculated by the graphical method of the previous section. Also note that given this linear model it is simple to solve for *i* and *j.*

## 4 Applying insights from the theoretical model

To illustrate how to incorporate the insights gained from the theoretical model in practice we will re-analyse a published economic evaluation comparing two eye care services in Zambia: cataract surgery and refractive error correction, compared to the situation before using these services (Griffiths et al., 2014). This study concluded that both eye-care services would be highly cost-effective in Zambia as the ICER estimates for these estimates are below the often mentioned threshold of GDP per capita (which equalled US$ 1160 for Zambia): the estimated cost effectiveness equalled $259.15 per QALY gained for cataract surgery and $375.00 per QALY gained for refractive error correction. Expected QALY gains per patient equalled 0.36 for cataract surgery and 0.19 for refractive error correction. Given the crisis in the workforce shortage, this study reported that only 45% of cataract patients and 36% of refractive error patients received the expected care. However, costs that were allocated to those who attended the facilities but did not receive the expected care were excluded in the calculations. By definition, this leads to an underestimation of ICERs. Therefore, we recalculated the ICERs using the information regarding costs and health effects as presented by the authors. As such, in contrast to the original calculations, the total overhead costs are reallocated to only those patients who received the care to obtain the actual costs per actually treated patient. The adapted cost estimates lead to a higher cost-effectiveness ratio: changing from $259.15 to $369.17 per QALY gained for cataract surgery and from $375.00 to $631.58 per QALY gained for refractive error correction.

In the absence of any health care input constraints the decision rules of cost effectiveness would imply that first all patients who need cataract surgery would be treated before any patients who refractive error correction are to be treated. In this study, 77 patients received the cataract surgery and 41 patients a refractive error correction. However, almost 100 patients who needed cataract surgery did not receive cataract surgery. One of the reasons mentioned in the study is a shortage of workforce of delivering cataract surgery implying a health care input constraint. To explore the impact of this constraint for ICER estimates and optimal decisions in this example, we first estimated total costs and total labour costs for each of the two interventions based on the data presented in the study. Although the information presented in the study did not allow a precise estimation of labour cost per patient for the two interventions, we made a rough estimate of $20 per patient for cataract surgery and $10 per patient for the refractive error correction. These labour costs per patient translate into $55.6 (20/0.36) and $52.6 (10/0.19) labour costs per QALY gained. Assuming that optimal use of the constrained resource was being made in the trial on which the economic evaluation was based and plugging in these values in equation (9) yields a $\frac{\lambda_{x}}{\lambda }\;$ ratio of 89.7 and adjusted ICER estimates of $5355 for both eye-care interventions. These values are substantially higher than the original ICERs and suggest that the GDP per capita threshold would be too lenient for interventions that require even a low amount of labour. Note that the ICER estimate of $5355 can also be used as a threshold level of cost-effectiveness to which new eye-care interventions could be judged.

To better understand the impact of health care input constraints we then estimated how much health could have been gained if there were no shortages of skilled labour. Based on the number of patients the total budget spent on these two interventions is $15,153.- of which $1950.- is spent on labour and total QALYs gained equal 35.5. Given that cataract surgery is more cost effective and that not all patients who need cataract surgery are treated because of labour shortages, we then assumed that not more than $1950.- can be spend on labour. To quantify the impact of HRC we estimated how many QALYs would be gained if the total budget ($15,153.-) could be spend freely (ignoring the labour budget constraint of $1950.-). In that situation all the budget would be spend on cataract surgery (treating 114 patients) resulting in 41.05 QALYs gained. The health care input constraint in this example results in a loss of almost 6 QALYs (more than 10% lower health gains). Furthermore, the labour budget constraints also implies that expansion of the health budget without relaxing the labour constraint should result in an expansion of refractive error corrections at the expense of cataracts. Obviously, this is a short-term optimal solution as an optimal long-term solution would be to invest in a more skilled labour force.

## 5 Conclusions and recommendations

In this paper, we illustrated how the presence of health care input constraints influences the decision rules of cost effectiveness and offered practical solutions to re-analyse and interpret cost effectiveness studies in such circumstances. In standard cost effectiveness analyses, it is implicitly assumed that opportunity costs are equal everywhere in the health care sector and that it does not matter from what health care inputs resources are drawn from when ‘old’ technologies are replaced by ‘new’ technologies. However, in the presence of health care input constraints, it is important to realize from where health care resources are being drawn from. Consequently, conventional ICERs need be adjusted by taking into account that some costs will have larger displacement effects in terms of health forgone than other costs. In our examples, we showed using how to adjust ICERs from published studies in the presence of a health care input constraint in specific cases. These examples revealed that optimal decisions may depend crucially on such adjustments.

A limitation of our theoretical model is that we assumed our spending function to be additive, whereas in some cases it might be more realistic to assume that health care inputs are multiplicative. However, adding non-linear constraints to the model would complicate the analysis substantially. It should be noted that the additivity assumption is in line with standard cost effectiveness analyses which looks at changes in spending at the margin where additivity might suffice. In this paper, we stressed the impact that health care input constraints have on decision rules. However, also the estimation of costs and benefits of technologies might be influenced by health care input constraints. Currently, many cost effectiveness studies ignore such constraints when estimating costs and benefits of health care technologies (Vassall et al., 2016). While this offers relevant information of the efficiency of new technologies under ideal circumstances (which might be achievable in the long run), it does not give insights into how these technologies might work out in practice. Given that usually investments are needed in order to reach those ideal circumstances, it might not always be cost-effective to operate technologies under ideal circumstances. Related literature in the area of economic evaluation deals with issues related to constraints with respect to implementation of health care technologies in practice (Fenwick et al., 2008, Hoomans and Severens, 2014). Note that although our starting point was that of incremental economic evaluations targeted at decision makers who might not have the discretion to relax health care input constraints, such constraints itself signal that the health system needs strengthening in terms of health care inputs (Morton et al., 2016).

In the presence of health care input constraints conventional ICERs presented in economic evaluations become less informative for decision makers. Therefore, less focus should be placed on only estimating and presenting this conventional ICER. Rather, it is better to present results in a manner that aligns with relevant constraints. For instance, in the context of human resource constraints it is important that results of economic evaluations need to be presented so that decision makers can see how much human resources costs are required to gain QALYs. In specific cases, depending on the availability of data, it is possible to adjust conventional ICERs. Key for our proposed adjustments of conventional ICERS is that constraints are expressed in monetary costs rather than units of health care inputs such as number of doctors or nurses. As such, our proposed approach is less flexible than a more general mathematical programming approach. But, it is easier to understand and is a more natural extension of cost effectiveness results. However, even in the absence of suitable data for ICER adjustments, the issue of how health care input constraints might affect optimal decisions needs to be discussed properly in applied cost effectiveness analysis. Crucial is that opportunity costs and thresholds are constraint specific and that opportunity costs are higher (and thresholds lower) for the constrained health care input. Furthermore, a scarcity of estimates of threshold levels of cost effectiveness should not be an excuse to ignore health care input constraints when making policy recommendations. Rather, we advise to make use of rules of thumb. For instance, new technologies that are cost-effective compared to currently funded programs but require lots of skilled labour should not automatically be funded. Vice versa, new technologies that are not cost-effective compared to currently funded programs but require less skilled labour can be attractive. Before applying such rules of thumb, one must start by identifying health care input constraints by focusing on a criterion like the presence of skilled labour. Tools like to OneHealth Tool promoted by the WHO for strategic health planning may help in identifying health care input constraints as this tool makes use of several coverage indicators that identify ‘bottlenecks’ in the delivery of health care services (Avenir Health, 2012).

Although the insights from our theoretical model apply to any health care input shortage the relevance of accounting for health care input constraints in decision rules is most easily demonstrated for the cost effectiveness analysis of task shifting. The motivation for investigating the cost effectiveness of task shifting usually is the presence of human resource constraints and the idea that highly skilled/trained labour (which is scarce) can be made more productive if some of their tasks can be shifted to other less scare labour forces (often also less skilled) (Fulton et al., 2011). However, published literature on task shifting considers task shifting only worthwhile in case it does not result in health losses and also results in cost savings (Griffiths et al., 2014, Mdege et al., 2013, Vaughan et al., 2015). So the standards appear to be higher for these type of interventions than for standard health care technologies while our model indicates the reverse: interventions that free up constrained health care inputs should be judged against a higher threshold rather than a lower threshold.

We conclude that it is possible to use the results of cost effectiveness studies in the presence of health care input constraints. In the presence of health care input constraints, the issue of opportunity costs expressed in health foregone becomes even more important as they differ between health care inputs. We showed how to adjust conventional ICERs for such differences in opportunity costs between health care inputs in specific cases. These adjustments are crucial as applying the default decision rules of cost effectiveness analysis without such adjustments might lead to health losses.

## Funding

This study is financially supported by the Bill and Melinda Gates Foundation. The funding agreement ensured the authors' independence in designing the study, interpreting the data, writing, and publishing the report. The authors would like to thank Karl Claxton, Talitha Feenstra, Jeremy Lauer, Paul Revill, Mark Sculpher, Anna Vassall and all participants of the International Decision Support Initiative (www.idsihealth.org) workshop in York for their helpful comments and suggestions on draft versions of this paper.
